# Spatial Characterization of Microbial Communities on Multi-Species Leafy Greens Grown Simultaneously in the Vegetable Production Systems on the International Space Station

**DOI:** 10.3390/life11101060

**Published:** 2021-10-09

**Authors:** Mary E. Hummerick, Christina L. M. Khodadad, Anirudha R. Dixit, Lashelle E. Spencer, Gretchen J. Maldonado-Vasquez, Jennifer L. Gooden, Cory J. Spern, Jason A. Fischer, Nicole Dufour, Raymond M. Wheeler, Matthew W. Romeyn, Trent M. Smith, Gioia D. Massa, Ye Zhang

**Affiliations:** 1 Kennedy Space Center, Amentum Services, Inc., LASSO, Merritt Island, FL 32899, USA; christina.l.khodadad-1@nasa.gov (C.L.M.K.); anirudha.dixit@nasa.gov (A.R.D.); aashelle.e.spencer@nasa.gov (L.E.S.); gretjmldo@gmail.com (G.J.M.-V.); jennifer.l.gooden@nasa.gov (J.L.G.); cory.j.spern@nasa.gov (C.J.S.); jason.a.fischer@nasa.gov (J.A.F.); 2 Kennedy Space Center, Utilization and Life Sciences Office, NASA, Merritt Island, FL 32899, USA; nicole.dufour@nasa.gov (N.D.); raymond.m.wheeler@nasa.gov (R.M.W.); matthew.w.romeyn@nasa.gov (M.W.R.); trent.m.Smith@nasa.gov (T.M.S.); gioia.massa@nasa.gov (G.D.M.)

**Keywords:** veggie, ISS, microbiome, lettuce, mizuna, phyllosphere, rhizosphere, microgravity

## Abstract

The establishment of steady-state continuous crop production during long-term deep space missions is critical for providing consistent nutritional and psychological benefits for the crew, potentially improving their health and performance. Three technology demonstrations were completed achieving simultaneous multi-species plant growth and the concurrent use of two Veggie units on the International Space Station (ISS). Microbiological characterization using molecular and culture-based methods was performed on leaves and roots from two harvests of three leafy greens, red romaine lettuce (*Lactuca sativa* cv. ‘Outredgeous’); mizuna mustard, (*Brassica rapa* var *japonica*); and green leaf lettuce, (*Lactuca sativa* cv. Waldmann’s) and associated rooting pillow components and Veggie chamber surfaces. Culture based enumeration and pathogen screening indicated the leafy greens were safe for consumption. Surface samples of the Veggie facility and plant pillows revealed low counts of bacteria and fungi and are commonly isolated on ISS. Community analysis was completed with 16S rRNA amplicon sequencing. Comparisons between pillow components, and plant tissue types from VEG-03D, E, and F revealed higher diversity in roots and rooting substrate than the leaves and wick. This work provides valuable information for food production-related research on the ISS and the impact of the plant microbiome on this unique closed environment.

## 1. Introduction

Future space missions and habitats will likely include crop production systems designed to provide a fresh, nutritious component to the crew diet. Understanding the complexities of plant growth systems designed with the unique challenges of space exploration missions and the associated plant microbiome is essential for sustaining the health of crop systems as well as the human consumer [1,2]. Molecular biology methods to characterize whole microbial communities through omics approaches have led to the understanding of organism and habitat-specific microbiomes. Plants and their associated microbiota exist in a complex association with important functions critical to plant growth and health. Microbes are recruited and assembled on or in plant tissues and organs from reservoirs such as the soil, rhizosphere, and phyllosphere [3,4,5,6]. Soil microbial communities are highly diverse and are horizontally transferred to the root where, at the rhizosphere, a dynamic and complex ecosystem develops [7,8,9,10,11,12]. Initial soil microbiome composition can influence plant pathogen interaction and disease resistance [13]. A plant’s phyllosphere hosts a diverse community of microbes that can also influence plant health and physiology [14,15,16,17]. Plant colonizing microorganisms are affected not only by abiotic environmental conditions such as CO_2_ concentration, temperature, spectral radiation, and humidity, but specific host leaf surface characteristics [14,18,19]. Studies have shown inter-plant species variability in the phyllosphere microbiome [14,16,20] and microbial colonization of the phylloplane is dependent on transmission of organisms from the environment such as aerosols, soils or other plant growth medium, and animal contact [21,22,23,24]. Conversely, it has been demonstrated that plants can influence the microbiome of the indoor built environment significantly contributing to the bacterial and fungal abundance and diversity on the surrounding surfaces [23]. 

**Figure 1:**
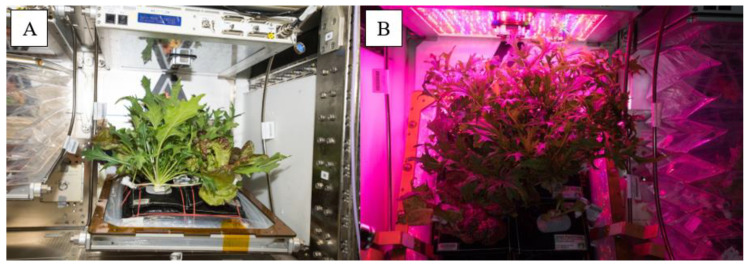
(**A**) VEG-03D (SN 002) with ambient light and bellows down. (**B**) VEG-03E (SN 001) with bellows lowered and plant base plate raised to a more vertical position and VEG-03F (SN 002) partially visible on the right of VEG-03E with bellows raised.

The International Space Station (ISS) is an inimitable, closed environment that has been continuously inhabited by astronauts starting with Expedition 1 in November of 2000. Unlike terrestrial, built environments, the exchange of microbes from outside the ISS is infrequent and primarily limited to the exchange of international crews after months of residency on the space station, along with re-supply missions. Since 2014, plants, primarily leafy greens, have been grown in the NASA Veggie hardware which are small growth chambers on the ISS (ORBITEC now Sierra Space., Madison, WI, United States) [25,26]. Fifteen technology demonstrations directed by NASA to grow a variety of crops have been completed to date and currently there are two Veggie units (Serial number (SN) 001 and Serial number 002) in operation on ISS. The Veggie chambers (Figure 1) utilize a fan to circulate the air from outside of the chamber throughout the plant growth area facilitating an exchange of airborne microbes between the ISS atmosphere and the chamber interior. Air drawn from the ISS cabin flows from the bottom of the plant canopy to the top of the growth chamber volume. Air filtration is not present apart from screens to filter out large particles within the Veggie facility before entering the cabin [27]. Environmental conditions in which ISS grown crops have been cultivated are unlike terrestrial counterparts, in addition to an absence of gravity and natural convection. Carbon dioxide levels can reach approximately 7 to 10 times higher than ambient earth atmosphere in the closed atmosphere of the ISS, temperatures are typically between 18 °C and 23 °C and humidity is low (40–50%), however temperature and humidity can be higher inside the Veggie chamber. A collapsible, transparent bellows contains the plant growing area and any plant debris. During the on-orbit operation, the plant crops are tended, harvested, and consumed by the crew and are intended to provide a nutritional supplement to their diet. At the completion of a harvest, all plant growth containers, known as plant pillows, are discarded, or stored frozen for return to Earth and the bellows and other surfaces are cleaned with disinfectant wipes in preparation for the next crop. 

Microbial analyses using culture-based and culture-independent methods of Veggie-grown crops and growth chamber components have been completed to provide baseline data to ensure the microbiological safety for consumption by the astronauts. Subsequently, an in-depth characterization of bacterial and fungal communities associated with three crop grow-outs of red romaine lettuce that may impact astronaut and plant health has been reported [28].

Plants grown in Veggie are susceptible to abiotic factors inherent in microgravity plant cultivation such as water stress and humidity control. Without careful control and monitoring, the environmental conditions within Veggie can promote undesirable levels of microbial growth. *Zinnia hybrida*, a flowering plant grown in Veggie in 2015–2016 became infected with the plant opportunistic pathogen *Fusarium oxysporum* after failure of the ventilation system led to an increase in humidity inside the chamber [29]. The source of the fungus was not determined definitively, however *F. oxysporum* has been isolated on surfaces on the ISS [29,30,31,32,33]. Furthermore, *F. oxysporum* was among the fungal isolates identified from leaves and roots from two out of three Veggie demonstrations growing red romaine lettuce [28]. The introduction of microbes from the air, water, or human interaction onto the plant surfaces of an edible crop potentially establishes a risk to the consumer. Crew safety and health is of paramount consideration when supplying a fresh food source through Veggie crop production and the characterization of the microbial communities in Veggie furthers the assurance that these crops are appropriate and safe for human consumption.

The VEG-03D, E, and F series of technology demonstrations discussed in this work are the first mixed crop use of the Veggie facility with the growth of three leafy greens, red romaine lettuce (*Lactuca sativa* cv. ‘Outredgeous’); mizuna mustard, (*Brassica rapa* var *japonica)*; and green leaf lettuce, (*Lactuca sativa* cv. ‘Waldmann’s Green’). 

In addition to the demonstration of a mixed cultivar approach and continuous harvest using two Veggie systems, the objective of this study was to characterize and compare the bioburden and composition of the microbial communities within the Veggie plant growth units at different locations including above ground plant tissues, roots of 3 plant cultivars, Veggie facility surfaces, and plant pillow materials. We identified through culture- based and culture-independent methods dominant and shared genera of bacteria and fungi in plants, growth substrate, and Veggie facility surfaces. This study demonstrated simultaneous multi-species leafy green growth and the influence of the plant leaf and root associated microbial communities across plant species and Veggie growth chamber components.

## 2. Materials and Methods

The VEG-03D, VEG-03E, and VEG-03F technical demonstrations aboard ISS present a mixed, multi-crop study with the inclusion of a “cut-and-come-again” component where plants were partially harvested and then re-grown to yield more produce for a given the amount of resources and to determine effects on the harvest. A total of four harvests were conducted for each grow-out, with the second and half of the fourth/final harvest preserved for later microbial analysis, and the remaining harvests consumed by astronauts. The leafy greens included were red romaine lettuce (*Lactuca sativa* cv. ‘Outredgeous’); mizuna mustard, (*Brassica rapa* var japonica); and green leaf lettuce (*Lactuca sativa* cv. ‘Waldmann’s Green’). Three separate grow-outs were conducted in the two Veggie production systems on ISS with initiation in September 2017 (VEG-03D) and February 2018 (VEG-03E and F). VEG-03E and F were grown three days apart in different Veggie units to test the concept of sustainable cultivation and continuous harvest for provision of fresh produce (Table 1).

### 2.1. Seed Preparation 

Seeds for each crop were surface sanitized using a chlorine gas fuming method as described by Massa et al. [34]. Briefly, seeds were batch sanitized by adding 0.5 mL HCL to 30 mL bleach in a 250 mL container by placing an open Petri dish containing the seeds into the container, without submersion, and sealing. Seeds were sanitized for one hour, removed from the container and allowed to off-gas overnight. Seed germination tests were performed to verify seed viability. Sanitization was confirmed for bacteria on Trypticase Soy Agar plates (TSA) (BD, Franklin Lakes, NJ, USA) and mold/fungi were confirmed on Inhibitory Mold Agar (IMA) (BD, Franklin Lakes, NJ, USA). Plates were incubated at 30 °C for 24–48 h for observation of bacterial growth and up to 120 h for fungal growth. 

### 2.2. Pillow Assembly, Layout, and Processing

**Figure 2:**
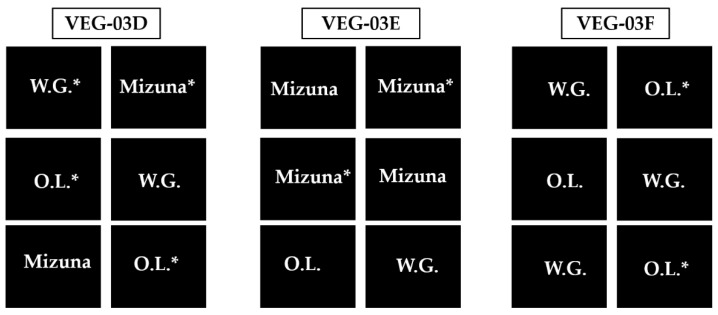
Veggie technical demonstrations on ISS (VEG-03D, VEG-03E, and VEG-03F) showing the pillow and plant layout for each. The ‘*’ indicates pillows returned from ISS for analysis. ‘OL’ is ‘Outredgeous’ red romaine lettuce; ‘WG’ is ‘Waldmann’s Green’ leaf lettuce; ‘Mizuna’ is mizuna mustard greens.

All pillows were assembled at Kennedy Space Center, FL and prepared for flight as described in detail by Massa et al., 2017 [34]. Briefly, the Veggie pillows contained 250 mL of autoclaved, porous, arcillite substrate with controlled release fertilizer (Nutricote 18-6-8, type 100, Florikan ESA, Sarasota, FL, USA) mixed at 7.5 g/L dry substrate. Two surface sanitized seeds were attached with guar gum to germination wicks in each plant pillow. The mixed crop pillow layout for each study is shown in Figure 2. 

The Veggie production system is a small plant growth chamber. Designed and built by ORBITEC (Madison, WI, US) to grow crops in space, it is equipped with LED lighting, a passive watering system and a fan to generate airflow [25,26]. The SN 002 system was launched to the ISS in 2014 aboard Space X CRS-3, while the SN 001 unit was launched aboard Orbital ATK OA-7 in 2017. A HOBO (honest observer by onset) data logger (Onset, Bourne, MA, US) was placed in the Veggie units used for VEG-03D and VEG-03E to record humidity, dew point, and temperature data (Figure S1). HOBO data were not collected from VEG-03F.

### 2.3. On-Orbit Operations

On-orbit operations for VEG-03D, E, and F were similar to previous Veggie tech demo missions [28,35] with the exception of (1) 160 mL water injection for initiation; and (2) the root mat was installed without being filled with water to mitigate potential overwatering observed in previous missions. Because these growth tests were conducted by the crew autonomously using their own time, there were no ground control experiments conducted. 

During VEG-03D operation, a fan anomaly occurred, causing a rise in humidity to 100% for approximately 25 days inside the Veggie facility and condensation on the bellows surface (Table S1). During the growth tests, wipes containing Pro-San (Microcide, Sterling Heights, MI, USA), an organic acid-based food surface sanitizer had been used one or two times to sanitize any obvious fungal growth on the surface of pillows and/or on the threads that hold the pillows together. The crew followed the Veggie tech demo protocols to harvest samples for analyses and to sanitize the surface of fresh leaves with Pro-San wipes before consumption. Pictures were taken for the Veggie team to evaluate plant health and other potential issues, and watering dates and volumes were documented.

### 2.4. Surface Sample Collection 

Surface swab samples were collected by the crew from 8 different sites within or on the Veggie facility using self-contained sterile swabs (Becton Dickinson, Franklin Lakes, NJ, USA), three from the interior bellows, one from the upper one-third of the bellows, one in the middle, and the third from the lower portion, three plant pillow top surfaces, one from the internal fan screen, and one from the fan outlet vent ports. After sample collection, the swabs were returned to the sterile container and stored frozen at −80 °C in the Minus Eighty Laboratory Freezer on ISS (MELFI).

### 2.5. Sample Processing 

**Figure 3:**
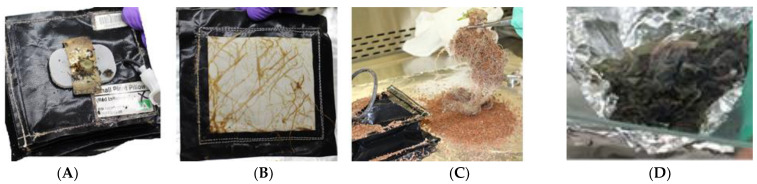
Veggie pillow after return from ISS; (**A**) top and (**B**) bottom, (**C**) root and arcillite substrate sample collection, (**D**) frozen leaf tissue sample.

Leaves from the second and final harvests, swabs and selected plant pillows were maintained at −80 °C in MELFI until returned to Earth. Upon return all samples were stored at −80 °C until processing. Plant leaf samples and swabs were removed from the freezer and processed immediately. Pillows were thawed at 4 °C overnight. Substrate, wicks, and roots were removed from each pillow and divided for microbiological and molecular analyses (Figure 3).

### 2.6. Microbiological Analysis

Leaf and pillow samples were placed into 50 mL centrifuge tubes with sterile phosphate-buffered saline (PBS) and 3–4 mm sterile glass beads, weighed then shaken for 2 cycles at 5 m/s for 30 s each on the Omni BeadRuptor (OMNI, Kennesaw, GA, USA). Swabs were placed into tubes containing sterile PBS with 0.3% Tween 80 and vortexed at high speed for 30 s before plating. All samples were diluted with PBS and plated in duplicate onto TSA for bacteria and IMA for fungi. Plates were incubated at 30 °C for 48 h for TSA and 72–120 h for IMA and enumerated. Colony phenotypes were selected from each sample and isolated on the respective agars. Bacterial colonies were identified using the Biolog Micro ID System (Biolog, Hayward, CA, USA). The MicroSEQ 16S rDNA sequencing kit was also utilized to identify bacterial isolates while fungal and yeast colonies were identified using the MicroSEQ D2 LSU rDNA kit for fungi (Thermo Fisher, Waltham, MA, USA). DNA was extracted from isolates using Prepman System for bacteria or the Qiagen Powerlyzer Soil Kit for fungi. All MicroSeq sequencing was completed on the ABI 3130 Genetic Analyzer (Thermo Fisher, Waltham, MA, USA). Bacterial and fungal DNA sequences were identified using the MicroSeq ID Software V2.0 (Bacterial Library, 2009 and Fungal Library 2011) followed by NCBI Basic Local Alignment Search Tool (BLAST).

Petrifilms were also used to identify and enumerate *Escherichia coli*/coliform and *Staphylococcus aureus* for microbial food safety screening (3M, St. Paul, MN, USA). Petrifilms were incubated at 35 °C for 24 h per manufacturer’s guidelines and any colonies positive for *E. coli*/coliform and *S. aureus* were enumerated and re-isolated. Re-isolated colonies were identified using Biolog GEN III plates. To screen for *Salmonella*, 1 mL of sample extract was added to an additional volume of buffered peptone water (BPW) and incubated at 35 °C for 24 h. A 1 mL aliquot was then transferred into 5 mL of Rappaport-Vassiliadis (RV) broth or Tetrathionate broth (Thermo Fisher, Waltham, MA, USA) and incubated for an additional 24 h at 35 °C. The broth cultures were then streaked onto selective media for *Salmonella* and incubated at 35 °C for 24–48 h [36]. 

### 2.7. Molecular Community Analysis

Upon processing, components for all samples were placed in RNA*later* for preservation and frozen at −20 °C until DNA isolation was completed using the Qiagen Microbial Cell DNA Isolation Kit (Qiagen, Inc., Carlsbad, CA, USA) following manufacturer’s protocol. DNA was quantified with the QUBIT 2.0 and the double stranded high sensitivity DNA assay (Invitrogen, Inc., Grand Island, NY, USA) then diluted to 0.5 ng/µL for downstream polymerase chain reaction (PCR). PCR was completed for each sample in triplicate with barcoded primers from the V4 region of the 16S rRNA gene for bacteria and barcoded primers for the internal transcribed spacer (ITS) for fungi. Unique barcodes were assigned for both forward and reverse primers (300 nM) to multiplex samples [28,37,38]. The PCR cycling conditions in a Bio-Rad C-1000 thermocycler included enzyme activation at 95 °C for 5 min, followed by 30 cycles of 95 °C for 1 min, annealing at 58 °C for 1 min and 72 °C for 2 min for extension. A final 10 min extension at 72 °C concluded the PCR. PCR products were cleaned with the Min-Elute PCR Cleanup Kit to remove excess primers and dNTPs (Qiagen, Carlsbad, CA, USA), and sequenced as an equimolar library spiked with 10% PhiX DNA [28]. Sequencing was completed as paired end on the Illumina MiSeq Next Generation Sequencer (NGS) [28]. FastQ sequences were identified to the genus level using the RDP/GreenGenes and SILVA (bacteria) and Unite (fungal) databases. 

### 2.8. Data Analysis

Data from microbiological counts (log transformed) were compared using a one-way ANOVA followed by Tukey’s multiple comparisons test using GraphPad Prism version 8.0.0 for Windows (GraphPad Software, San Diego, CA, USA). Alpha diversity was determined using the Shannon Diversity Index and beta diversity of community sequencing was determined with Bray–Curtis dissimilarity [39] using a one-way ANOVA (QIIME 2.0) [40]. Venn diagrams were created using Venny 2.1. [41].

## 3. Results

### 3.1. Plant Germination and Growth

VEG-03D was designed with a mixed horticulture layout of two pillows for each cultivar to examine the interaction of all three crop types (Figure 2). In Veg-03D, we found that the more rapidly growing mizuna plants overshadowed adjacent lettuce plants, causing watering and accessibility issues for plant maintenance. Therefore, for the VEG-03E and F growth tests, the layout was adjusted for easy access, observation, and plant maintenance (Figure 2).

The overall emergence rates for mizuna and red romaine lettuce were 91.6% and 83.3%, respectively. Green leaf lettuce seeds averaged 66.6 percent germination (75% in VEG-03D vs. 62.5% in VEG-03E/F) with two stunted seedlings. After thinning, one healthy green leaf lettuce seedling failed to develop further, resulting in only two out of four pillows with healthy green leaf lettuce plants in VEG-03E and F compared to two out of two pillows with healthy green leaf lettuce plants in VEG-03D. Except for the two green leaf lettuce plants, all other plants appeared healthy. Tip burn was observed later during the grow-outs in red romaine lettuce plants. In addition, older mizuna leaves also turned yellowish with a dry edge. All these signs occurred after the 30–31-day harvest, and increased with time, especially for mizuna, indicating a possible lack of nutrients with the age of the plant. No signs of disease were found in these plants. Water usage was relatively constant with about one liter consumed per plant per 30 days (Table S1). Mizuna required a slightly higher water volume to maintain hydration and plant health compared to both lettuces. These experiments were performed by the crew in a semi-autonomous manner; the water volume was adequate for maintaining plant health, but may not have been optimal, and may have varied among the crew members who tended these plants. 

### 3.2. Bacterial and Fungal Counts on Leaves

**Figure 4:**
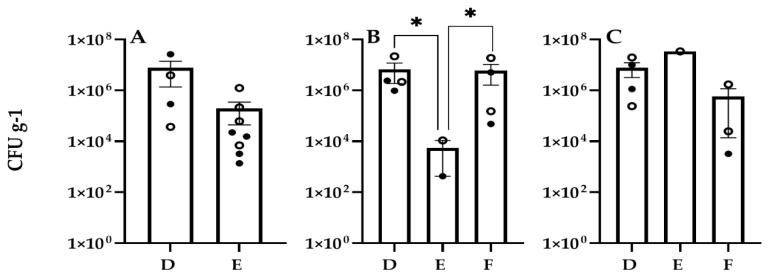
Bacterial counts (CFU on TSA per gram of frozen tissue) on leaves from plants grown in VEG-03D, E, and F for three crops. (**A**) mizuna, (**B**) red romaine lettuce, (**C**) green leaf lettuce. Open circles represent counts from the first harvest, closed circles the final harvest. Bars represent the mean and error bars are standard error of the mean. Statistical differences (*p* < 0.05) are indicated with an asterisk.

Aerobic plate counts (APC) of bacteria on leaves from Veg-03D, E, and F harvests of the three leafy greens were in the range of 4.3 × 10^2^ (red romaine in VEG-03E) to 3.5 × 10^7^ colony-forming units (CFU) per gram (g) of leaf tissue. Average counts on mizuna leaves from both harvests grown in VEG-03D and E were not significantly different from each other, ranging from 1.4 × 10^3^ to 2.6 × 10^7^ CFU/g. The average counts on green leaf lettuce across experiments D, E, and F were 7.9 × 10^6^, 3.5 × 10^7^, and 5.9 × 10^5^ CFU/g, respectively. Only one harvest was completed on the green leaf lettuce plant from E. Red romaine lettuce grown in VEG-03E had lower counts than those harvested from D and F (*p* ≤ 0.05). Average bacterial counts from red romaine lettuce leaves from D, E, and F were 6.9 × 10^6^, 5.7 × 10^3^, and 6.1 × 10^6^ CFU/g^,^ respectively (Figure 4).

**Figure 5:**
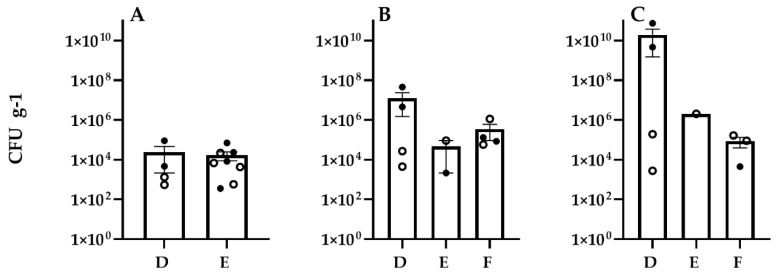
Fungal counts (CFU on IMA per gram of frozen tissue) on leaves from plants grown in VEG-03D, E, and F for three crops. (**A**) mizuna, (**B**) red romaine lettuce, (**C**) green leaf lettuce. Open circles represent counts from the first harvest, closed circles the final harvest. Bars represent the mean and error bars are standard error of the mean.

Yeast and mold counts from the leaves grown in VEG-03D were lowest on average on mizuna, 2.4 × 10^4^ CFU/g followed by 5.1 × 10^6^ CFU/g on red romaine lettuce leaves and green leaf lettuce with the highest average counts at 9.8 × 10^9^ CFU/g, however the difference between plant types was not significant. The fungal counts were higher on the second harvest leaves from all three plant types grown in VEG-03D (Figure 5). The fan anomaly may have contributed to this trend, which was not found in VEG-03E and F. All screening tests for potential food borne pathogens, *E. coli*, *S. aureus* and *Salmonella* sp. showed negative results.

### 3.3. Bacterial and Fungal Counts on Pillow Components

Plant pillows were investigated for bacterial and fungal counts from the wicking material, the roots, and the substrate inside the pillow. Figure 3 illustrates a plant pillow after the plant had been harvested and before sampling including the wicking material (Figure 3A) that initially holds the seed and wicks water from the substrate to the germinating seedling, and the substrate and roots after removal from the pillow (Figure 3C). Four pillow samples from VEG-03D (2 red romaine, 1 mizuna and 1 green leaf lettuce), 2 from VEG-03E (mizuna) and 2 from VEG-03F (red romaine) were processed for analysis. 

**Figure 6:**
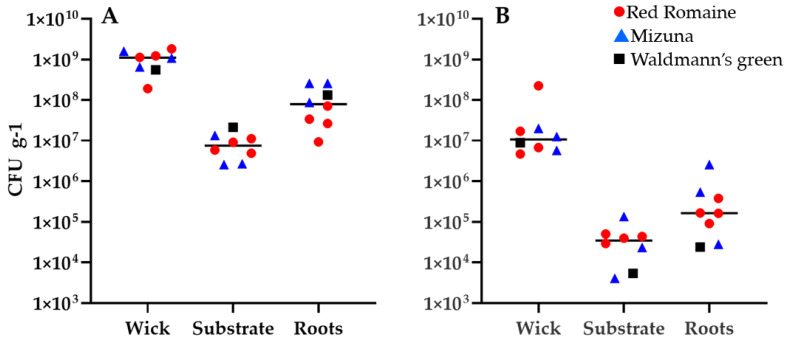
Bacterial (**A**) and fungal (**B**) counts (CFU per gram) on plant pillow components from VEG-03D, E, and F. Bars represent the median.

Bacterial counts on the wicking material and arcillite substrate were not influenced by plant type or Veggie facility (SN 001 or SN 002) used in the tech demo (Figure 6A). The wick samples had APC counts ranging from 1.9 × 10^8^ to 1.8 × 10^9^ CFU/g. Average counts from the substrate samples (*n* = 3) ranged from 2.6 × 10^6^ to 2.2 × 10^7^ CFU/g. CFU/g values on the red romaine lettuce roots grown in VEG-03F were the lowest of the root bacterial counts, 9.3 × 10^6^ and 2.6 × 10^7^ compared to 3.4 × 10^7^ and 7.1 × 10^7^ CFU/g from the red romaine lettuce roots grown in VEG-03D. Bacterial counts from mizuna and green leaf lettuce roots were all higher than the red romaine lettuce counts. Two mizuna plant roots grown in VEG-03E yielded 8.7 × 10^7^ and 2.5 × 10^8^ CFU/g while the mizuna roots grown in VEG-03D had the highest bacterial counts of the mizuna, 2.6 × 10^8^ CFU/g. Only one sample of roots from the green leaf lettuce grown in VEG-03D was returned for analysis, yielding 1.3 × 10^8^ CFU/g bacteria. 

Fungal counts are illustrated in Figure 6B. The lowest and highest counts on the wicks 4.7 × 10^6^ to 2.2 ×10^8^ CFU/g were from the 2 red romaine pillows from VEG-03D. Fungal counts were highest in mizuna roots and substrate from the same VEG-03E pillow with the highest bacterial counts, however, the lowest fungal counts were from the VEG-03D pillow that also had similarly high bacterial counts, indicating no correlation.

### 3.4. Bacterial and Fungal Counts on Surface Samples

Swab samples were collected after the harvest was complete but before wiping down of the interior bellows with disinfectant wipes. While each sample site is a different location, distinct sample sites are the same across the three tech demos. Bacteria were recovered and cultivated from all 8 sample sites from VEG-03D, 3 out of 8 samples in VEG-03E and 5 out of 8 in VEG-03F (Table 2). Fungi were recovered from 5, 3, and 7 samples out of 8 from VEG-03D, E, and F, respectively. Bacteria and fungi were recovered from the fan screen and at least two of the pillow surfaces across all the experiments (Table 3). VEG-03E was run in SN 001 Veggie facility, a unit that was just installed and had not been used before on ISS, had the fewest locations with recovery of bacteria and fungi. VEG-03D and F were run in the same Veggie SN 002 facility, the older Veggie on ISS. Bacterial counts (CFU/swab) on surfaces from VEG-03D ranged from 3.5 × 10^1^ on the fan screen to 6.0 × 10^4^ on the pillow surface. Several samples from VEG-03E were below the detection limit with the exception of the fan screen and two pillow samples. VEG-03F had the highest CFUs on the pillow surfaces ranging from 1.3 × 10^5^ to 4.0 × 10^6^/swab. Fungi from VEG-03D samples ranged from below detection to 4.5 × 10^3^ CFU per swab. The highest fungal load, 4.0 × 10^6^ CFU/swab was found on samples from VEG-03F on a pillow sample (Table 2).

### 3.5. Cultivated Bacteria and Fungi Isolate Identification

Cultivation and recovery of the bacteria and fungi present in all samples was dependent on survival during sample storage as well as growth medium and incubation conditions and represents a fraction of the taxa present, however community sequencing verified the presence of each of the cultivated bacterial and fungal genera. A total of 30 bacterial genera were isolated on TSA and identified from VEG-03D, E, and F plant, pillow, and surface samples (Table 3). Several genera were isolated from samples from all three Veggie sample sets including *Burkholderia*, *Chryseobacterium*, *Leifsonia*, *Microbacterium*, *Pseudomonas* and *Staphylococcus*. More genera were recovered and identified from VEG-03D (20) than from E or F, and eight of the 30 isolates were unique to VEG-03D samples (Table 3). 

The fungi, *Aspergillus* spp., *Fusarium oxysporum, Penicillium* spp. *and Rhodotorula* spp. where ubiquitous across samples from all three tech demos (Table 4). *Exophiala* spp. was identified in only one sample from VEG-03E, the substrate, and swab samples from E and F recovered *Purpureocillium lilacinum.* All fungi, except the *Purpureocillium* isolated only from swab samples, were verified by community sequencing. Because swab samples yielded no DNA, we were not able to verify the presence of *Purpureocillium.* An additional 26 genera of fungi were identified in samples using community sequencing of the ITS region (Table S5).

### 3.6. Microbial Diversity

Alpha and beta diversity represent the variation within and between samples. Alpha diversity of VEG-03D, E, and F, determined by the Shannon Index, ranged between 0.1 (very low) and 2.0 (Tables S2–S4). Arcillite substrate and root tissue had the highest diversity indices followed by the wick, then leaf tissue. Leaf tissue for all plant types had the lowest alpha diversity. Alpha diversity for the swabs taken for the three experiments fell between 0.8 and 1.4. The distribution as determined by the Pielou Evenness Index measurement indicated the genera present were not evenly distributed with values ranging from 0.3 for substrate to 0.1 for leaf tissue. This trend was consistent between all three experiments (Tables S2–S4).

**Figure 7:**
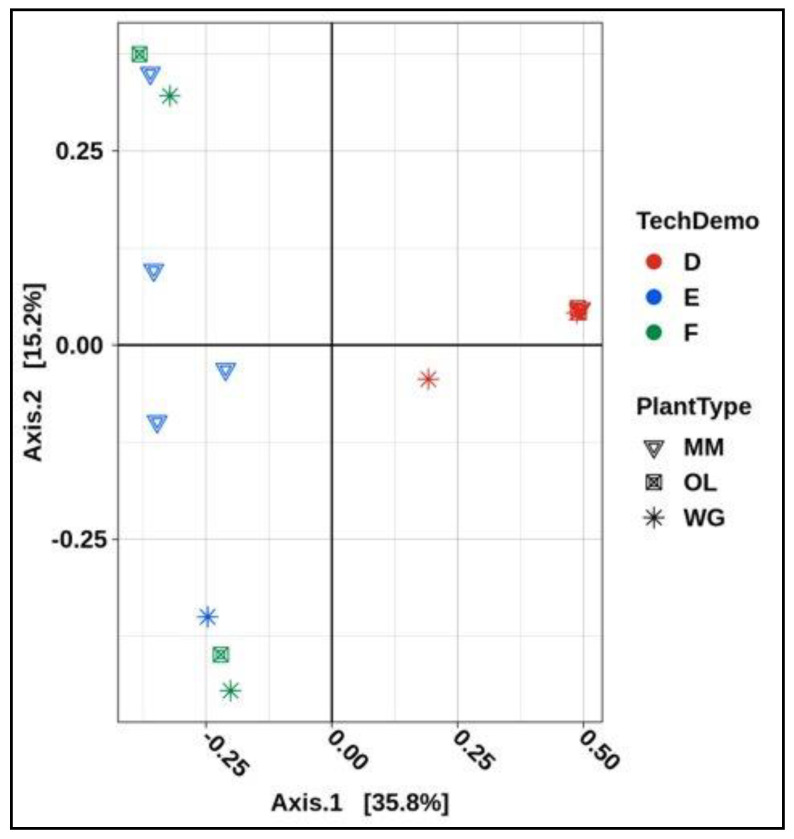
PCoA plot represents the plant leaf tissue returned from VEG-03D, E, and F on the ISS. Plant type MM is mizuna mustard, OL is ‘Outredgeous’ red romaine lettuce and WG is ‘Waldmann’s Green’ leaf lettuce.

**Figure 8:**
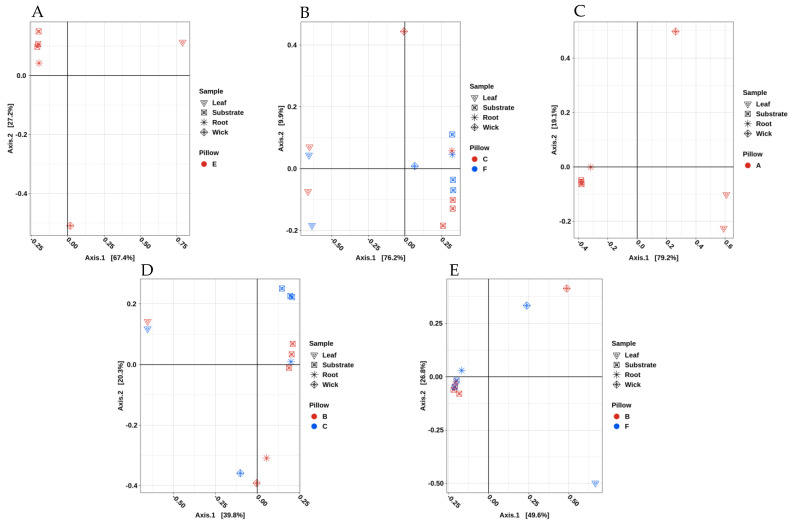
PCoA plots represent the plant and pillow components for VEG-03D, E and F tech demonstrations performed on ISS. (**A**) is VEG-03D mizuna mustard, (**B**) is red romaine lettuce, (**C**) is green lettuce, (**D**) is VEG-03E mizuna mustard and (**E**) is VEG-03F red romaine lettuce.

Beta diversity as determined between samples with the Bray–Curtis calculation in QIIME 2.0 was conceptualized as PCoA plots (Figure 7 and Figure 8A–E). Beta diversity was calculated between all leaf tissues returned from ISS (Figure 7 and Table S6). Just over 50% of the variation between plant types could be explained in the 2 axes of the PCoA (Figure 7). Plants from VEG-03D presented a visual clustering of leaves for all plant types with the exception one green lettuce leaf demonstrating similarity of the microbial communities. VEG-03E and F grown later do not cluster tightly indicating potential diversity differences. The corresponding Adonis statistics provides a permutation statistic of the analysis of variance which provides an R^2^ value indicating the % variance explained by each group. The lower the R^2^ value the more similar the samples. The R^2^ value for all plant types (0.397) indicated that approximately 40% of the variation is derived from the influence of the tech demo (*p* = 0.001) versus only 10% (0.101) was due to the plant type. The influence of the plant type was not significant.

Beta diversity was also compared among the plant types to include the plant leaf and root and the Veggie pillow substrate and wick. Each crop type was considered separately within the three experiments (Figure 8). VEG-03D contained the following: one pillow with mizuna mustard (Figure 8A), two pillows with red romaine lettuce (Figure 8B) and one pillow with green leaf lettuce (Figure 8C). The PCoA of the three plant types with their associated pillow components in VEG-03D captured a high percentage of the variation within a single axis, with 67%, 76%, and 79%, respectively.

The PCoAs for VEG-03D indicate that the green leaf lettuce leaf microbiome was more dissimilar from the root, wick, and substrate. However, the root and substrate were similar. Approximately 99% of the variation accounted for within the pillow sample set was due to the sample type itself (Figure 8C and Table S6). The additional two crops grown in VEG-03D displayed the same trends with root and substrate microbiomes more similar while the plant leaf microbiomes were dissimilar containing a greater amount of variation (97% and 84% for mizuna and red romaine lettuce, respectively) (Figure 8 and Table S6). In all pillow samples, the wick, which did not cluster with root and substrate, appeared to be more variable.

The variation captured for the VEG-03E and F experiments was lower than seen in VEG-03D with approximately 40% of the variation explained in the first principle component for VEG-03E (Figure 8D) and approximately 50% for VEG-03F (Figure 8E). In these two experiments, the wick again appeared to have the greatest variance from other components. Leaf, root, and substrate were more similar. The R^2^ values for VEG-03E indicated 63% of the variation was due to the sample type, whereas in VEG-03F, 77% of the variation was attributed to the sample type. All comparisons were significant (*p* < 0.05) (Figure 8 and Table S6). 

### 3.7. Community Characterization 

**Figure 9:**
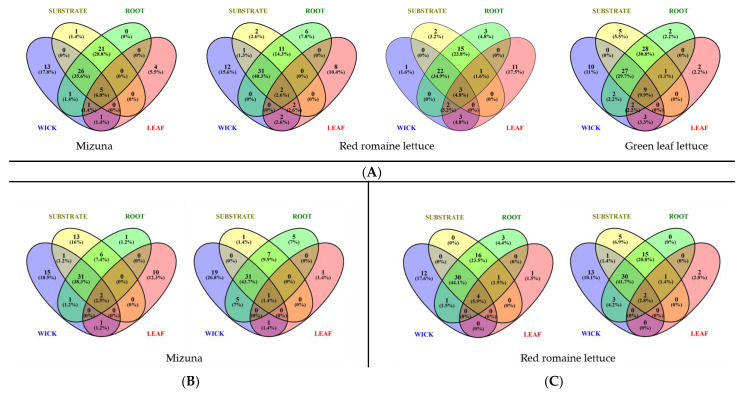
Venn diagrams revealing unique and shared genera across the 3 tech demos and crop types. (**A**) VEG-03D samples, (**B**) VEG-03E samples, (**C**) VEG-03F samples. Diagrams depict plant and corresponding pillow components (wick, substrate, and root tissue). Numbers are the number of genera with greater than 50 sequencing reads. The % is the percent total reads. Venn diagrams were completed with Venny v 2.1.

Community characterization of the pillow components and plant tissue (leaf, root, substrate, and wick) was analyzed at the genus level to determine unique and shared genera (Figure 9). There were few genera common to all four components in each of the three technical demonstrations, (VEG-03D, E, F) and ranged between one and nine genera. VEG-03D green leaf lettuce plant and pillow samples had the highest number of shared genera with nine (Figure 9A). In all three tech demos the wick component revealed a high number of unique genera between 2–19; the highest number of unique genera occurring in VEG-03E mizuna mustard pillow wicks (Figure 9B). 

Figure 9A illustrates a high number of genera shared between wick, substrate, and root tissue in VEG-03D with 26 in mizuna, 22 and 31 in red romaine lettuce, and 27 in green leaf lettuce. The number of shared genera among VEG-03E components was higher for mizuna mustard at 31 and VEG-03F had 30 shared genera between wick, substrate, and root tissue (Figure 9C). 

Root tissue had few unique genera, but in all crops the root had high numbers of shared genera with substrate (6–28) and between root, substrate, and wick (22–31) which included more than 40% of the genera present (Figure 9). Leaf tissue had a variable number of unique species with VEG-03D having the highest with 11 in red romaine lettuce, followed by 10 in mizuna in VEG-03E. 

Overall, characterization of genera in the four plant and pillow components revealed few unique species in each of the components but indicated a higher number of shared species especially among root, substrate, and wick (Figure 9).

**Figure 10:**
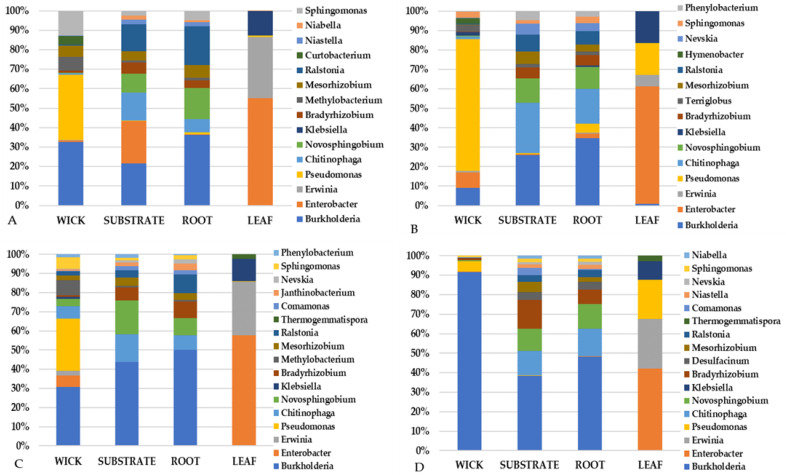
VEG-03D technical demonstration. The top 15–17 genera for the Veggie pillows and corresponding plants as determined by 16S rRNA gene sequencing. (**A**) is Mizuna mustard; (**B**) is green lettuce and (**C**,**D**) are red romaine lettuce. Percent is the percent total reads of the top genera.

**Figure 11:**
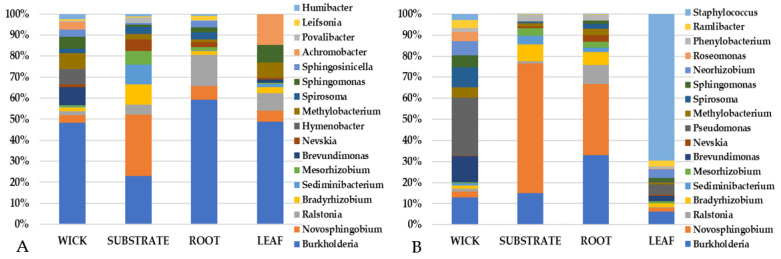
VEG-03E technical demonstration. The most abundant 15 genera for the two Veggie pillows and corresponding mizuna mustard leaf tissue. Percent is based upon the abundance of sequencing reads.

**Figure 12:**
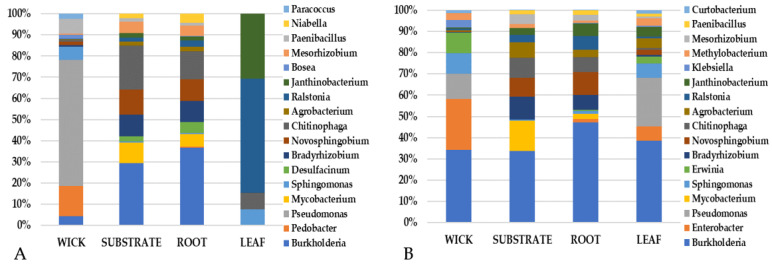
VEG-03F technical demonstration. The most abundant (17) genera for the Veggie pillows and corresponding ‘Outredgeous’ red romaine lettuce leaf tissue grown on the ISS and determined by 16S rRNA gene sequencing. Percent is based upon the abundance of sequencing reads.

A closer inspection of the top community constituents revealed that variations in the bacterial makeup between pillow sections (wick, substrate, and root) differed from the leaf tissue (Figure 10, Figure 11 and Figure 12). VEG-03D which included analysis of all three leafy green crops grown yielded several bacterial constituents seen in all plant types (Figure 10). These included *Burkholderia*, *Enterobacter*, *Erwinia* and *Pseudomonas*. However, there were fewer numbers of genera in leaves than in the root, wick, or substrate, which is not unexpected. There were more similar constituents in the wick, substrate, and root with *Burkholderia* and *Novosphingobium* identified in each component for all crops (Figure 10, Figure 11 and Figure 12). The wick material appeared to present a higher variation in genera than did root or substrate.

The VEG-03E samples and pillows returned from ISS were mizuna mustard (Figure 11) while VEG-03F were red romaine lettuce (Figure 12). Again, all samples contained *Burkholderia* with some samples containing up to 50% of the total abundance of sequencing reads. VEG-03E mizuna mustard pillow substrate and root tissues contained higher levels of *Novosphingobium* than seen in the VEG-03D tech demo. *Staphylococcus* was present in mizuna leaf from one VEG-03E pillow accounting for a large percent of the 15 listed and present in small fraction in the wick although was not detectable in the root or substrate. The wick material had a higher level of variability in the bacterial genera detected (Figure 11). The VEG-03F tech demo comparing red romaine lettuce plant and pillow samples showed less variation in the bacterial genera in the wick and leaf compared to VEG-03D and E. *Pseudomonas* which was prevalent in most wick materials in VEG-03D, E and F could also be detected in leaf tissue (Figure 10, Figure 11 and Figure 12). *Ralstonia,* and *Pseudomonas* were also present in all samples from all three tech demos. (Figure 10 and Figure 12). Several genera were present in VEG-03D (Figure 10) and F (Figure 12) but not in E. The genera *Enterobacter*, *Chitinophaga*, *Novosphingobium* and *Bradyrhizobium* are a few that were identified in all of the Veg-03D samples as well as some of the VEG-03F samples. In VEG-03D *Methylobacterium* was identified in red romaine lettuce and mizuna while *Janthinobacterium* was identified in red romaine.

## 4. Discussion

The Veggie plant growth units deployed on the ISS have provided a platform to grow leafy greens for astronaut consumption since 2014. This series of tech demonstrations was the first time that mixed cropping was tested in Veggie, with three leafy greens. Plant growth was successful, however, there may have been an influence on growth based on plant type and position in the Veggie. It was observed in VEG 03-D that the mizuna canopy shaded the adjacent red romaine lettuce plant possibly limiting exposure to light and effecting maintenance. Germination was lower with the green leaf lettuce resulting in fewer and smaller plants. Mixed cropping could also have an impact on the transfer of plant-specific phyllosphere microbiota between crop types [14,16,20].

Microbiological food safety of Veggie-grown crops is assured by routine culture-based pathogen screening and bacterial and fungal enumeration on crops grown in ground analogs as well as samples returned from ISS. The bacterial and fungal count data and pathogen screening performed on the plants grown in VEG-03D, E, and F indicates from a microbial food safety perspective, the crops are low risk to the crew. With approximately 10% of a microbial community identified with culturable methods, sequencing of both bacterial and fungal constituents provides additional identification of the community microbiota [42]. These data allow for the identification of the non-culturable microbes including the identification of beneficial microbes and microbes of concern and the confirmation of the negative culture-based screening results for *E. coli* and *Salmonella*. Many of the genera isolated and identified from the Veggie plant phyllosphere are present in terrestrially grown leafy greens. Several surveys of leafy greens revealed that *Pseudomonas* species dominate the phyllosphere. A study done on two types of leafy greens, rocket salad and lettuce, showed that *Pseudomonas*, was the most abundant genera present, followed by *Pantoea*, *Arthrobacter* and *Acinetobacter* [14]. In this study, *Pseudomonas* sp. was identified by culture independent methods on all samples except one mizuna plant in VEG-03E. *Burkholderia* was identified in all plant samples by culture and culture independent methods. This is in contrast with other surveys of field grown leafy greens where *Burkholderia* is not a dominant member of the core microbiome [14,15,16]. However, *Burkholderia* was found on both ISS grown and ground controls in an earlier study done on Veggie grown red romaine lettuce [28].

Bacterial counts (CFU/g) from field and hydroponic grown lettuce have been reported in the range of 4 × 10^4^ to 2 × 10^8^ and fungal counts (CFU/g) between 8 × 10^2^ and 2 × 10^6^ [43,44,45,46,47,48,49,50,51]. The bacterial and fungal counts on leaves of plants grown in VEG-03D, E, and F were in the range of the reported values for farm or market produce counts of similar type. However, when compared to the analysis of red romaine lettuce from previous Veggie tech demos [28], the bacterial and fungal counts per gram of red romaine lettuce leaf tissue from VEG-03D and F were higher. As with previous ISS grown leafy greens [28,51] an increase in fungal counts was observed in some of the samples with repeated harvests especially in VEG-03D. VEG-03A and VEG-03D were run in the same Veggie facility (SN 002) and during plant growth, in both cases, the humidity was higher than other grow-outs. Humidity is a factor that can influence the microbial density and community in the phyllosphere and the surface of the leaf. It has been demonstrated that higher humidity and leaf wetness increases fungal abundance on plants including fungal plant pathogens [29,52,53]. 

The characterization of the spatial distribution of microbiota on the Veggie facility surfaces, plant growth pillows, and multi-species plant tissues was performed to elucidate possible sources and routes of transfer of microbes around the Veggie facility and plants. Generally, surface sample microbial count data showed low counts on the interior bellows surfaces, and below detection level counts mostly in VEG-03E. After harvest, the bellows are cleaned thoroughly by the crew with disinfectant wipes, perhaps minimizing microbial proliferation. The pillow surfaces had the highest counts of all surfaces sampled and are in close proximity with the moist wicking material, the plant and any plant debris which could facilitate inoculation and growth. Bacteria and fungi were recovered from the fan screen in all cases. The genera of bacteria and fungi cultured and identified have also been identified in studies to monitor the microbial profiles of the ISS. *Staphylococcus*, *Bacillus*, *Pantoea*, *Pseudomonas*, *Aspergillus* and *Penicillium* were all identified in an examination of ISS air filters and dust samples [54]. Several of the same genera from the previous study were isolated and identified from the ISS vacuum cleaner bag, and also included *Paenibacillus*, *Methylobacterium*, *Klebsiella*, and *Azospirillum* [55]. In addition, studies done on the microbial communities associated with surfaces on the ISS identify many of the same genera identified from the Veggie surface samples collected in this study [30,32]. We compared bacterial genera from Avila-Herrera et al. [56] comprehensive characterization of ISS surface and crew samples with the Veggie facility surfaces and leaves characterized in the current study and found 250 genera shared between the Veggie facility surfaces and ISS samples. The leaves, Veggie surfaces and ISS samples had 47 common genera suggesting microbial transfer and colonization between these locations (Figure S2).

Beta diversity analysis of all plants grown in the three tech demos resulted in a tight clustering of plants from VEG-03D with the exception of one green lettuce leaf demonstrating similarity of the microbial communities within VEG-03D. VEG-03E and F grown later do not cluster tightly indicating potential diversity differences (Figure 8). These differences could be the result of many factors, including the different configuration of the three crops in each Veggie, the environmental conditions present at the time of the grow-out, i.e., temperature, CO_2_ and humidity, crew interaction, microbes present in the surrounding air, and irrigation water. Previous studies on field-grown leafy greens have shown that seasonal and temporal variation exists on the phyllosphere microbial communities, effected by leaf age, weather, location and soil type [14,15,57].

Unlike terrestrial agriculture, a sanitized, soilless substrate is used in Veggie to support root growth. It is aseptically packed into sterile “pillows” along with controlled release fertilizer [34]. The seeds are surface sanitized before planting, removing most epiphytes from the seed surface, and the water used to hydrate the plant pillows is relatively clean water from the ISS potable water system [35,58]. Consequently, the horticultural practices employed in Veggie may limit the sources of microbiota contributing to the development of the plant microbiome. As a whole, the phytomicrobiome is shaped and recruited by the plant from its associated habitat [3,4,6,10,59]. Rhizosphere microbiomes assemble through the selection of soil microorganisms by the production of plant root exudates [60,61,62]. The Veggie soilless substrate is essentially microbe free until the introduction of water, seed germination and exposure to the ISS environment. Water and air from the ISS have been monitored by NASA for many years and two genera commonly identified from the potable water are *Burkholderia* and *Ralstonia* [58,63]. The dominant bacterial genera in Veggie samples analyzed in this study, *Burkholderia*, was found across plant types and Veggie facility materials in both culture and molecular assays. The genus *Burkholderia* contains over 60 known species and was abundant in many of the Veggie samples, primarily in the pillow substrate, root tissue, and wicks, but in very low abundance or was absent in leaf tissue indicating the genus may have been introduced through the water source or possibly was present in the seed. *Burkholderia* has been identified as a potential, beneficial plant-associated microbe, however some species are implicated in human opportunistic infections [21,64,65] *Ralstonia*, also found in ISS water, was identified in community sequencing across all plant type roots, wicks, and substrate. Some species of *Ralstonia* have the potential to cause stress in plants or may be an opportunistic pathogen [66,67,68]. *Pseudomonas* sp., present in the top 15 genera in community sequencing in all samples but one mizuna plant and pillow from VEG-03E may be a saprophytic epi-endophyte on plant tissue [16,64,69,70,71]. It has been reported to be a potential promotor of plant growth by producing plant stimulating hormones as well as increasing disease resistance [71]. These demonstrations, D and F were completed in the same Veggie unit, SN 002, and therefore not surprising that similar microbes would be detected. Several genera were identified by sequencing in VEG-03D and F but not in E. The genera *Enterobacter*, *Chitinophaga*, *Novosphingobium*, and *Bradyrhizobium* are a few that were identified in all of the Veg-03D samples as well as some of the VEG-03F samples. *Chitinophaga* sp., works with certain fungal species as an endosymbiont to enhance nutrient uptake and growth [66,72]. The presence of *Chitinophaga* could be correlated with stress conditions and environmental disturbances [66,72]. In VEG-03D *Methylobacterium* was identified in red romaine and mizuna while *Janthinobacterium* was isolated in red romaine only. Recently it was reported that 4 strains of *Methylobacterium*, one a newly characterized strain, were isolated on the ISS with the Microbial Monitoring experiments [73]. *Methylobacterium* are involved in nitrogen fixation, phosphate solubilization, abiotic stress tolerance, plant growth promotion, and biocontrol activity against plant pathogens [74,75] and is a methylotrophic with the ability to utilize methanol produced by plants as a carbon source [76] *Janthinobacterium* is a soil bacterium with a wide range of metabolic activities, including antimicrobial properties and resistance to stressors such as UV radiation and cold temperatures providing a benefit to plants [77,78,79]. During the grow outs of VEG-03E and F, the genera *Nevskia* and *Spirosoma* were present in VEG-03E but not seen in VEG-03F. This could be due to a natural variation between mizuna and red romaine lettuce as each plant may have its own native microbiota, or it may have been introduced. These bacteria were detected in root and leaf samples from previous Veggie tech demos, VEG-01A, B and VEG-03A [28]. If the source of these bacteria is the water initially introduced into the soilless substrate and subsequently wicked to the seeds, this suggests possible mechanisms of transfer of water borne bacteria from the seed to the root or recruitment by the root from the surrounding substrate. An in-depth genomic analysis would have to be performed to elucidate possible sources of microbial transfer.

All the bacteria cultured and identified from root samples, *Burkholderia*, *Flavomonas*, *Leifsonia*, *Methylobacterium*, *Microbacterium* and *Rhizobium* were also identified in the wick samples. Presumably, the sources of these bacteria are the ISS water, the endophytic seed population, circulating air, and/or human interaction with the plants, therefore diversity of the root microbiome could be limited by the populations in these sources. Eleven of the 12 identified isolates found on the leaf samples were not isolated from the roots, in corroboration with the 16S rRNA analysis of community comparison which revealed no shared species between the leaf and root. In the culture-based assays, 6 of the leaf-isolated bacteria were also found on the wicks. Different parts of the plant, i.e., leaves and roots support distinct microbial communities [80,81]. Patterns of genera common across the substrate, root, wicks, and leaves suggest alternate modes of bacterial and fungal transmission. For example, the substrate, roots, and wicks share the highest number of genera for all pillow sample sets tested while the leaves share a higher number of genera with the wicking material (Figure 9). This suggests the possibility of horizontal transfer of bacteria from substrate to root or the converse. The substrate starts as a sterile matrix therefore the introduction of microbes could be primarily limited to the ISS potable water, microbes within the ISS, those harbored on the Veggie surfaces, or the endophytic seed microbiota. The seed microbiome of two of the three crops, red romaine lettuce and mizuna mustard, have been investigated at KSC in numerous studies since the inception of the Veggie technology demonstrations. The microbiome of both sanitized and unsanitized seeds indicated that in all seeds, abundance of microbial communities/genera was low and the genus *Thermogemmatispora* predominated, and then declined post-germination., In addition, *Trichodesmium* and some genera of cyanobacteria were detected along with low abundance of *Burkholderia*, *Acinetobacter*, and *Corynebacterium*. These latter three appear to persist in the plants after germination [82]. Very few genera are shared between the leaf and substrate and the leaf and root whereas the wick has more genera in common with the leaf than the substrate and root, indicating a vertical transfer from the above “ground” wick material and the shoot of the plant. Resident microbial populations on the leaf may be transported from the ISS environment, including the circulating air and human interaction. Human associated *Staphylococcus* was identified on the leaves, surfaces, and wicks from cultured isolates and for 16S community sequencing, it was the dominant bacteria among the top 15 identified in one VEG-03E mizuna leaf sample. Other potential human and plant associated bacterial genera identified were *Erwinia, Burkholderia*, and *Clavibacter.* Extensive genotyping of these isolates would be required to evaluate the strain and the ability to infect the host.

Four of the fungal species identified, *Aspergillus* spp., *Fusarium oxysporum*, *Penicillium* spp., *Rhodotorula* spp. were found in all tech demos. In previous studies of Veggie-grown plants, *Fusarium oxysporum* was not found on the first growth of lettuce or surfaces of Veggie from the tech demo designated VEG-01A but has since been isolated in all subsequent tech demos [28] (unpublished data). This suggests that once bacteria or fungi become established in Veggie units and the ISS environment, unless eliminated by the cleaning and maintenance protocols, they could persist as members of the facility microbiome and consequently the plant microbiome.

In conclusion, characterization of the microbiome of ISS plant growth systems and crops is critical, not only for near term assurance of crop food safety, but also for the understanding and development of sustainable crop systems for space exploration missions. Sources of external contamination, i.e., irrigation water, air, and human handling can be monitored and controlled. Strategies, such as the introduction of defined beneficial microbes to enhance plant growth and prevent human and plant pathogen invaders may need to be evaluated and implemented after careful study of the impact to these unique space agricultural systems.

## Figures and Tables

**Table 1:** Technical demonstration scheduling for VEG-03D, VEG-03E, and VEG-03F grown aboard ISS.

Tech Demo	Plant/Pillow Initiation	1st Harvest	2nd Harvest	3rd Harvest	Final Harvest	Return to Earth	Plant analysis	Pillow Analysis	Swab Analysis
VEG-03D (SN002)	9/26/17	10/27/17	11/3/17	11/13/17	11/23/17	5/5/18	5/14/18	7/10/18	6/29/18
VEG-03E (SN001)	2/6/18	3/8/18	3/14/18	3/27/18	4/6/18	8/30/18	10/4/18	11/27/18	11/19/18
VEG-03F (SN002)	2/9/18	3/11/18	3/17/18	3/30/18	4/9/18	8/3/18	10/4/18	11/27/18	11/19/18

**Table 2:** Bacterial and fungal CFU for surfaces based upon swab location for 2 Veggie facilities on ISS.

Sample	APC CFU/Swab			Fungi CFU/Swab		
	D	E	F	D	E	F
1-pillow	3.5 × 10^2^	2.1 × 10^4^	1.3 × 10^5^	<DL	8.0 × 10^3^	8.1 × 10^3^
2-pillow	6.0 × 10^4^	7.7 × 10^4^	4.0 × 10^6^	4.5 × 10^3^	1.6 × 10^4^	1.2 × 10^4^
3-pillow	3.5 × 10^2^	<DL	4.0 × 10^6^	4.9 × 10^3^	<DL	4.0 × 10^6^
4-bellows UL	1.5 × 10^3^	<DL	7.0 × 10^2^	<DL	<DL	2.5 × 10^2^
5-bellows MF	1.0 × 10^2^	<DL	<DL	1.4 × 10^2^	<DL	7.0 × 10^1^
6-bellows LR	1.1 × 10^3^	<DL	5.2 × 10^3^	1.8 × 10^2^	<DL	1.1 × 10^2^
7-fan screen	3.5 × 10^1^	4.1 × 10^4^	8.6 × 10^3^	3.5 × 10^1^	3.3 × 10^4^	5.1 × 10^3^
8-outlet	1.4 × 10^2^	<DL	<DL	<DL	<DL	<DL

**Table 3:** Bacterial isolates from VEG-03D, E, and F cultivated on TSA. VEG-03D wick, substrate, and root are from 2 ‘Outredgeous’ red romaine (OL) lettuce, 1 mizuna (M) and 1 ‘Waldmann’s Green’ leaf lettuce (WG). Leaves include 2 M, 2 OL and 2 WG. VEG-03E wick, substrate, and root are from M and leaves include 4 M, 1 OL and 1 WG. VEG-03F wick, substrate, and root are from OL. Plants include 3 OL and 3 WG.

Bacteria	Swab	Wick	Substrate	Leaf	Root
*Achromobacter*				E (WG, M), F (WG)	
*Acidovorax*		F			
*Amphibacillus*			D	D (Ol)	
*Bacillus*	D	D	D		
*Brevundimonas*		F	F		
*Burkholderia*		D, E, F	D, F	F (GL)	D, F
*Chryseobacterium*	E, F		D		
*Clavibacter*			D		
*Cupriavidis*			F		
*Curtobacterium*		F		E (WG), F (OL)	
*Enterobacter*	D				
*Fictibacillus*	F				
*Flavomonas*		D	D		D
*Gardinella*	D				
*Gordonia*			D		
*Klebsiella*		D, F		D (OL, WG)	
*Kocura*				D (WG)	
*Leifsonia*		D, E, F	D, E, F		D
*Methylobacterium*		D	D		D
*Microbacterium*	D, E, F	E, F	D, E, F	E (WGL), F (OL)	D, E, F
*Micrococcus*			D	E (M)	
*Nocardioidaceae*	F				
*Novosphingobium*	E, F				
*Paenibacillus*	E, F	E, F		E (M, OL), F (OL)	
*Pantoea*	D, F	F		D (WG)	
*Paracoccus*		F			
*Pectobacterium*				D (OL)	
*Pseudomonas*	D, E, F	D		D (M, WG, OL)E (WG), F (OL)	
*Rhizobium*		D, E			D, E
*Staphylococcus*	D, E, F	D, F		D (WG, M), E (M) F (WG)	

**Table 4:** Fungal genera identified by cultivation on IMA from the three VEG-03 tech demos.

Fungi	Swabs	Wick	Substrate	Leaf	Roots
*Aspergillus* spp.	D, E, F	D, E, F	D, E, F	D, E, F	D, E, F
*Fusarium oxysporum*	D, E, F	D, E, F	D, E, F	D, E, F	D, E, F
*Penicillium* spp.	F	D, F	D, E	D, E, F	
*Rhodotorula* spp.	D, F	D, E, F	D, E, F	D, E, F	D, E, F
*Exophiala* spp.			E		
*Purpureocillium lilacinum*	E, F				

## Data Availability

All sequences are available in NASA GeneLab with the following accession numbers: GLDS-412 and 10.26030/0jpd-te04; https://doi.org/10.26030/0jpd-te04; GLDS-413 and 10.26030/3p20-j525 https://doi.org/10.26030/3p20-j525; GLDS-414 and 10.26030/b487-6c39 https://doi.org/10.26030/b487-6c39.

## Supplementary Materials

The following are available online at https://www.mdpi.com/article/10.3390/life11101060/s1, Table S1: Water volume per plant, Table S2: Shannon index for VEG-03D alpha diversity, Table S3: Shannon index for VEG-03E alpha diversity, Table S4: Shannon index for VEG-03F alpha diversity, Table S5: Fungal genera, VEG-03 E and F, Table S6: Adonis tables for VEG-03D, E, and F. Figure S1: VEG-03D and E HOBO data, Figure S2: Venn diagram indicating shared genera between leaf samples, Veggie surface samples, and reported ISS samples.
