# Supplementary material for: Spatial Characterization of Microbial Communities on Multi-Species Leafy Greens Grown Simultaneously in the Vegetable Production Systems on the International Space Station
Source: Life (Basel). 2021 Oct 9;11(10):1060. doi: 10.3390/life11101060 (PMC8537831; doi:10.3390/life11101060)
Supplement: Supplementary file 1 [file life-11-01060-s001.zip › life-1377932-supplementary.pdf]

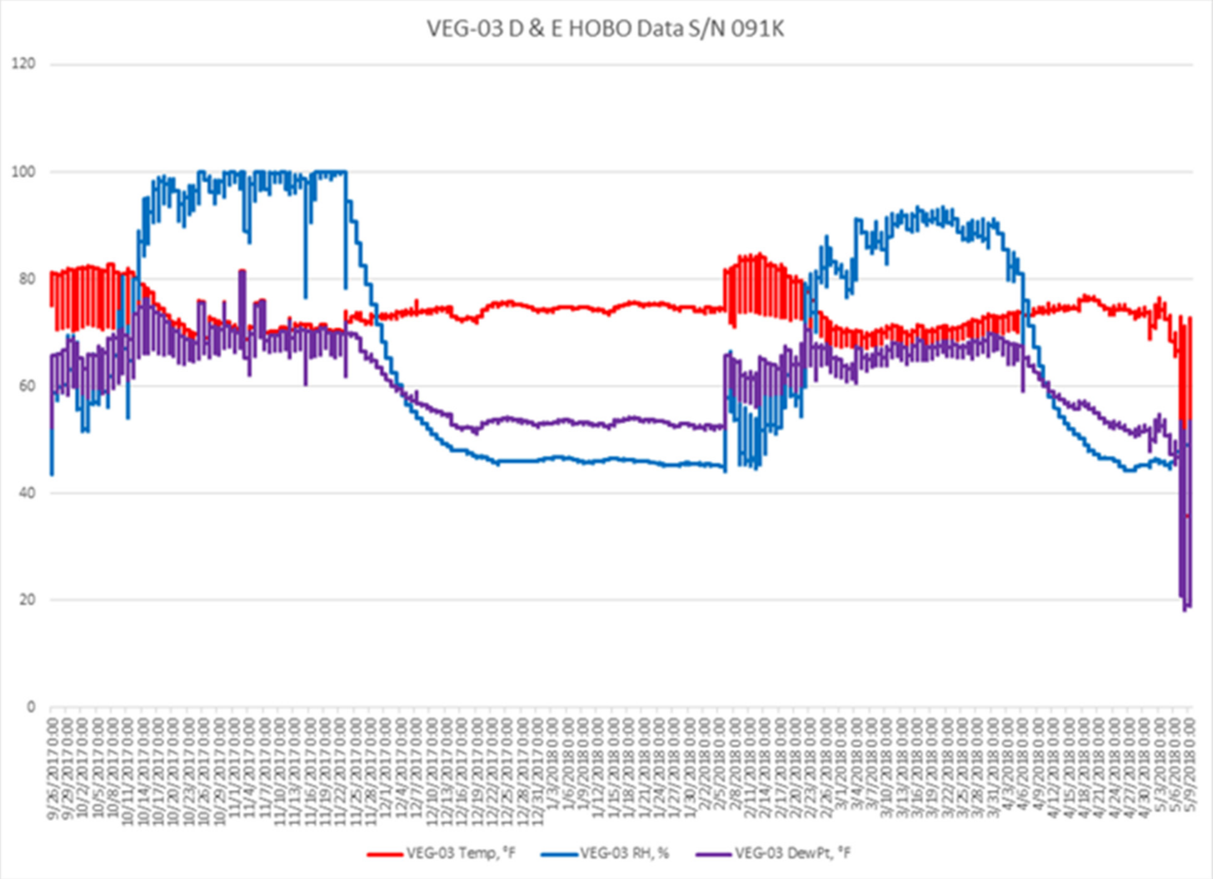

Figure S1. Veg-03 D and VEG-03 E HOBO data recording temperature and humidity. The first curves are VEG-03D, the second VEG-03E

Table S1. Water volume per plant from VEG-03 D, E and F combined.

	Total plant number	DAP30	SD	Final Harvest	SD
**Mizuna**	6	1143.3	122.9	2530.8	301.2
**Red Lettuce**	6	935.0	223.4	2162.5	475.1
**Green Lettuce**	4	943.8	44.8	2058.8	131.0

Table S2. Shannon Index for Alpha Diversity for each sample recovered in VEG-03D. Evenness is calculated by Pielou's Evenness Index. Community diversity includes richness (number of species) and evenness. Evenness is defined as how close in number each species is or another measure of biodiversity which quantifies how equal the community is numerically quantified. The range is from 0 to 1 where a value close to 0 means the numbers are uneven. The closer to 1 the numbers of each species are closer together.

VEG 03D			
**Sample**	**Shannon Index**	**# Species**	**Evenness**
V03DGreen05-S3	1.865	695	0.284998
V03DGreen05-S2	1.873	615	0.291671
V03DGreen05-S1	1.867	755	0.281738
V03DGreen05-W	0.99	501	0.159251
V03DGreen05-R	1.898	716	0.288727
Veg03DSw-8	0.996	661	0.153378
Veg03DSw-7	1.365	639	0.211303
Veg03DSw-6	0.757	377	0.127608
Veg03DSw-5	1.463	683	0.224163
Veg03DSw-4	0.889	768	0.133809
Veg03DSw-3	0.965	762	0.14542
Veg03DSw-2	0.925	736	0.140125
Veg03DSw-1	0.886	795	0.132668
FL-2F-Green-18	0.409	262	0.073451
FL-2E-Green-05	0.344	137	0.069919
FL-2D-Red-16	0.413	236	0.075588
FL-2C-Red-03	0.346	170	0.06737
FL-2B-Miz02	0.082	140	0.016594
FL-2A-Miz14	0.084	160	0.016551
FL-1F-Green-18	0.289	264	0.05183
FL-1E-Green-05	0.351	247	0.063709
FL-1D-Red-16	0.293	206	0.054994
FL-1C-Red-03	0.29	215	0.053997
FL-1B-Miz02	0.096	169	0.018714
FL-1A-Miz14	0.293	158	0.057875
Veg03DMiz2-W	1.47	422	0.243176
Veg03DMiz2-S3	1.945	540	0.309144
Veg03DMiz2-S2	1.735	405	0.288979
Veg03DMiz2-S1	0.696	437	0.114475
Veg03DMiz2-Root	1.959	530	0.312297
Red_0316_Wick	0.936	223	0.173103
Red_0316_Soil_3	1.767	344	0.302535
Red_0316_Soil_2	1.648	314	0.286639
Red_0316_Soil_1	1.653	220	0.306473
Red_0316_Root	1.666	325	0.288045
Red_0303_Wick	1.657	360	0.28151
Red_0303_Soil_3	1.791	404	0.29843
Red_0303_Soil_2	1.801	342	0.308665
Red_0303_Soil_1	1.666	413	0.276586
Red_0303_Root	1.853	484	0.299737

Table S3. Shannon Index for Alpha Diversity for each sample recovered in VEG-03E. Evenness is calculated by Pielou's Evenness Index. Community diversity includes richness (number of species) and evenness. Evenness is defined as how close in number each species is or another measure of biodiversity which quantifies how equal the community is numerically quantified. The range is from 0 to 1 where a value close to 0 means the numbers are uneven. The closer to 1 the numbers of each species are closer together.

	VEG 03E		
Sample	Shannon Index	# Species	Evenness
GMT_075_8_Plant	0.2	113	0.042307
23 GMT_075_7_Plant	0.121	81	0.027535
24 GMT_075_1_Plant	0.259	117	0.054387
25 GMT_075_13_Plant	0.115	80	0.026244
26 GMT_075_10_Plant	0.267	76	0.061652
GMT_096_8_Plant	0.154	119	0.032223
16 GMT_096_7_Plant	0.152	104	0.032728
17 GMT_096_1_Plant	0.2	120	0.041776
18 GMT_096_17_Plant	0.437	136	0.088954
19 GMT_096_13_Plant	0.192	159	0.037878
096_SN01_Wick_P	2.116	474	0.343439
46 096_SN01_Soil_3_P	1.523	333	0.262218
47 096_SN01_Soil_2_P	1.535	357	0.261155
48 096_SN01_Soil_1_P	1.332	238	0.243409
49 096_SN01_Root_P	1.973	419	0.326771
096_SN08_Wick_P	2.03	452	0.332042
41 096_SN08_Soil_3_P	1.835	347	0.313711
42 096_SN08_Soil_2_P	0.023	489	0.003714
43 096_SN08_Soil_1_P	1.964	452	0.321247
44 096_SN08_Root_P	1.804	283	0.31955
096_CFS_7_Swab	0.515	270	0.09199

Table S4. Shannon Index for Alpha Diversity for each sample recovered in VEG-03F. Evenness is calculated by Pielou's Evenness Index. Community diversity includes richness (number of species) and evenness. Evenness is defined as how close in number each species is or another measure of biodiversity which quantifies how equal the community is numerically quantified. The range is from 0 to 1 where a value close to 0 means the numbers are uneven. The closer to 1 the numbers of each species are closer together.

	**Veg 03F**		
**Sample**	**Shannon Index**	**# Species**	**Evenness**
GMT_078_WG_Plant	0.333	129	0.068521
21 GMT_078_Outred_Plai	0.411	91	0.091113
GMT_099_9_Plant	0.344	92	0.076076
12 GMT_099_6_Plant	0.176	83	0.039829
13 GMT_099_15_Plant	0.397	71	0.093134
14 GMT_099_12_Plant	0.97	110	0.206362
099_SN09_Wick_P	0.956	404	0.159296
28 099_SN09_Soil_2_P	1.616	509	0.259288
29 099_SN09_Soil_1_	1.647	378	0.277511
30 099_SN09_Root_P	1.781	372	0.300901
31 099_SN04_Wick_P	1.606	383	0.270005
32 099_SN04_Soil_3_P	1.471	338	0.252617
099_SN04_Soil_2_P	1.624	289	0.2866
34 099_SN04_Soil_1_P	1.553	464	0.252936
35 099_SN04_Root_P	1.809	397	0.302309
099_ML_2_Swab	1.242	176	0.24021
37 099_LL_3_Swab	1.381	266	0.247336
38 099_FAN_7_Swab	0.956	278	0.169876
39 099_BFU_5_Swab	0.343	138	0.069613

Table S5. Fungal genera determined by next generation sequencing using the ITS region. The list compiles plant, pillow and swab samples. VEG-03E/F were not de-multiplexed.

**Fungal Genera**	**VEG-03D**	**VEG-03E/F**
Alternaria spp	X	X
Aspergillus spp	X	X
Aureobasidium spp		X
Beauveria spp	X	X
Capronia spp		X
Cryptococcus spp		X
Cylindrocladiella spp		X
Cystobasidium spp		X
Didymella spp		X
Epicoccum spp		X
Erythrobasidium spp		X
Exophiala spp		X
Falciformispora spp		X
Fusarium spp	X	X
Gibberella spp	X	X
Kabatiella spp		X
Kockovaella spp		X
Malassezia spp	X	X
Microidium spp		X
Naganishia spp		X
Papiliotrema spp		X
Paramycosphaerella spp		X
Penicillium spp	X	X
Podospora spp		X
Pomotodes spp	X	X
Pseudoramichloridium spp		X
Rhodosporidiobolus spp	X	X
Rhodotorula spp	X	X
Stagonosporopsis spp		X
Teratosphaeria spp		X
Trichoderma spp		X

Table S6. Adonis tables

**All Plants**	**Df**	**SumsOfSqs**	**MeanSqs**	**F.Model**	**R2**	**Pr(>F)**
All Plant Types	2	0.584	0.292	0.974	0.101	0.448
TechDemo	2	2.287	1.144	3.813	0.397	0.001
PlantType:TechDemo	2	0.487	0.244	0.813	0.085	0.659
Residuals	8	2.399	0.300	NA	0.417	NA
Total	14	5.758	NA	NA	1.000	NA
**VEG-03D Mizuna Mustard Sample**	**Df**	**SumsOfSqs**	**MeanSqs**	**F.Model**	**R2**	**Pr(>F)**
Sample	3	1.662	0.554	38.349	0.975	0.01
Residuals	3	0.043	0.014	NA	0.025	NA
Total	6	1.705	NA	NA	1.000	NA
**VEG-03D Red Romaine Lettuce Sample**	**Df**	**SumsOfSqs**	**MeanSqs**	**F.Model**	**R2**	**Pr(>F)**
Sample	3	2.781	0.927	28.158	0.843	0.001
Pillow	1	0.053	0.053	1.624	0.016	0.212
Sample:Pillow	3	0.267	0.089	2.705	0.081	0.085
Residuals	6	0.198	0.033	NA	0.060	NA
**VEG-03D Green Leaf Lettuce Sample**	**Df**	**SumsOfSqs**	**MeanSqs**	**F.Model**	**R2**	**Pr(>F)**
Sample	3	1.651	0.550	93.653	0.989	0.005
Residuals	3	0.018	0.006	NA	0.011	NA
Total	6	1.669	NA	NA	1.000	NA
**VEG-03E Mizuna Mustard Sample**	**Df**	**SumsOfSqs**	**MeanSqs**	**F.Model**	**R2**	**Pr(>F)**
Sample	3	1.817	0.606	12.829	0.636	0.002
Pillow	1	0.254	0.254	5.388	0.089	0.004
Sample:Pillow	3	0.595	0.198	4.198	0.208	0.01
Residuals	4	0.189	0.047	NA	0.066	NA
Total	11	2.855	NA	NA	1.000	NA
**VEG-03F Red Romaine Lettuce Samples**	**Df**	**SumsOfSqs**	**MeanSqs**	**F.Model**	**R2**	**Pr(>F)**
Sample	3	1.584	0.528	17.330	0.775	0.003
Pillow	1	0.164	0.164	5.391	0.080	0.035
Sample:Pillow	2	0.204	0.102	3.344	0.100	0.105
Residuals	3	0.091	0.030	NA	0.045	NA
Total	9	2.044	NA	NA	1.000	NA

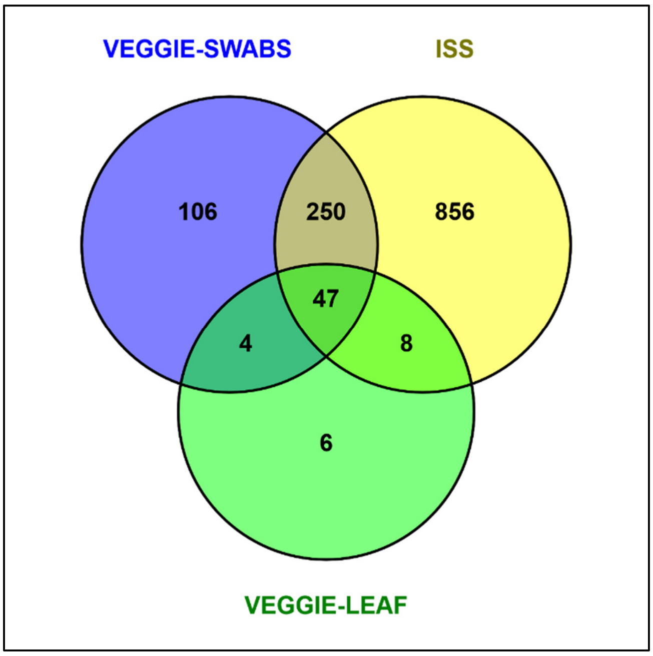

Figure S2. Comparison of bacterial genera from Veggie facility swabs, Veggie leaf samples and a data set from ISS surface and crew samples (reported by Avila-Herrera et al., 2020).
